# State-Level Household Energy Insecurity and Diabetes Prevalence Among US Adults, 2020

**DOI:** 10.5888/pcd21.240087

**Published:** 2024-08-29

**Authors:** Ryan Saelee, Kai McKeever Bullard, Jacob T. Wittman, Dayna S. Alexander, Darrell Hudson

**Affiliations:** 1 Division of Diabetes Translation, Centers for Disease Control and Prevention, Atlanta, Georgia; 2 Center for the Study of Race, Ethnicity & Equity, Brown School at Washington University, St. Louis, Missouri

## Abstract

The objective of this study was to examine the state-level association between household energy insecurity and diabetes prevalence in 2020. We obtained 1) state-level data on household energy characteristics from the 2020 Residential Energy Consumption Survey and 2) diagnosed diabetes prevalence from the US Diabetes Surveillance System. We found states with a higher percentage of household energy insecurity had greater diabetes prevalence compared with states with lower percentages of energy insecurity. Interventions related to energy assistance may help reduce household energy insecurity, mitigate the risk of diabetes-related complications, and alleviate some of the burden of diabetes management during extreme temperatures.

**Summary**

**What is already known on this topic?**

Energy insecurity is prevalent across the US and may be important for those with diabetes, who rely on stable energy access to reduce the impact of extreme temperatures.

**What is added by this report?**

Findings indicate that states with a higher prevalence of household energy insecurity had a higher prevalence of diagnosed diabetes, with the highest prevalence of both concentrated mainly among southern states.

**What are the implications for public health practice?**

Interventions and policies related to energy assistance may help reduce household energy insecurity, mitigate the risk of diabetes-related complications, and alleviate some of the burden of diabetes management during extreme temperatures.

## Objective

Climate change has led to increases in heat waves and cold spells, potentially worsening health outcomes among those with diabetes (1,2). Adverse physiologic responses to heat (eg, compromised vasodilation and sweating) and cold stress (eg, impaired vasoconstriction and brown tissue activity) may be factors driving the association between exposure to extreme temperatures and increased hospitalization and emergency department visits along with illnesses (cardiovascular disease, kidney disease, and hypertension) and death among those with diabetes (1–3). The use of residential heating and air conditioning is important for buffering against the adverse effects of extreme temperatures. However, evidence from previous research suggests that energy costs from residential heating and air conditioning are a significant burden to low-income households, which could subsequently contribute to inequalities in diabetes-related outcomes (4). In 2020, approximately 33.6 million of 123.5 million US households were considered energy insecure (ie, unable to adequately meet basic household energy needs) (5). Raising visibility at the state level of where those with energy insecurity and diabetes live may be informative for developing energy policies and interventions to meet the needs of those with diabetes. Thus, this study sought to examine the association between state-level household energy insecurity and diagnosed diabetes prevalence.

## Methods

We conducted cross-sectional analyses during August through October 2023 to examine the association between household energy insecurity and diabetes prevalence in 2020. We used data from the 2020 Residential Energy Consumption Survey (RECS), a nationally representative household survey that collects information on sociodemographic characteristics, energy use behaviors, and receipt of energy assistance (6). We used the Centers for Disease Control and Prevention’s US Diabetes Surveillance System to obtain 2020 state-level diagnosed diabetes prevalence estimates (7). We defined household energy insecurity as reporting any of the following in the past year: reducing or forgoing food or medicine to pay energy costs, leaving the home at what respondents felt were unhealthy temperatures, receiving a disconnect or delivery stop notice, and being unable — because of cost — to use heating equipment or air-conditioning equipment. We estimated weighted percentages and 95% CIs for any household energy insecurity, each of the 5 components, and those that had ever received energy assistance, overall and by state, accounting for the RECS sampling weights (6). Prevalence estimates were age-standardized to the 2000 US Census. To illustrate the relationship between age-standardized state-level prevalence of household energy insecurity and diagnosed diabetes, we categorized these variables into tertiles and created a bivariate choropleth map using R v4.3.2 package ggspatial (v1.1.8) (R Foundation) (8). We created a similar map of those who ever received energy assistance and diabetes prevalence. We used multivariable linear regression to assess the state-level association between age-standardized household energy insecurity and diagnosed diabetes prevalence, adjusting for state-level percentages of the population who are non-Hispanic White, experiencing poverty, and living in rural areas with data from the 2016 through 2020 American Community Survey (9–11) and the 2020 Housing and Demographic Characteristics file (12). We conducted these analyses in SAS v9.4 (SAS Institute Inc) and SAS-callable SUDAAN v11.0 (Research Triangle Institute).

## Results

**Figure 1:**
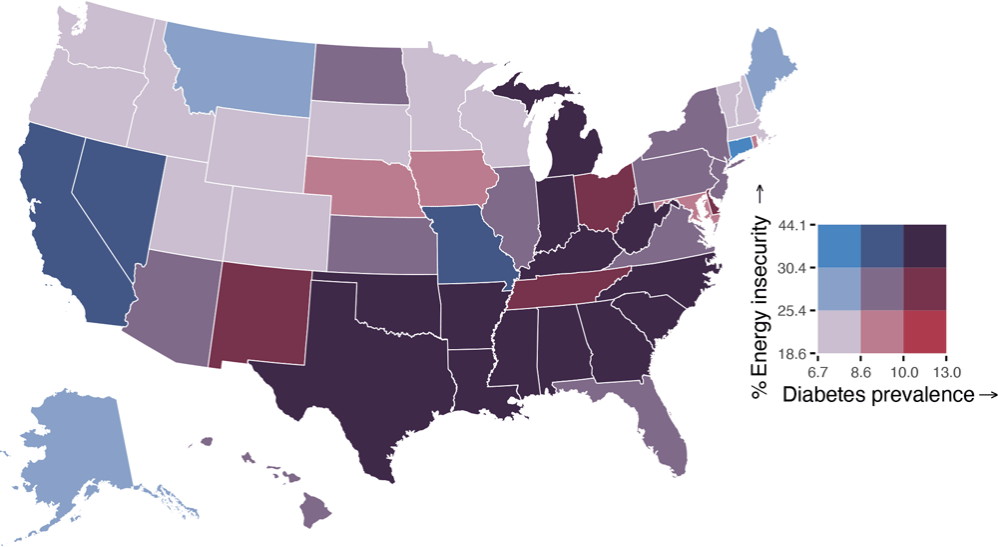
Bivariate map of the age-standardized percentage of any energy insecurity and diagnosed diabetes prevalence by US states, 2020. Note: Cutoffs for household energy insecurity and diabetes prevalence were established based on tertiles. Sources: 2020 Residential Energy Consumption Survey (RECS) (6); 2020 Centers for Disease Control and Prevention’s US Diabetes Surveillance System.

**Figure 2:**
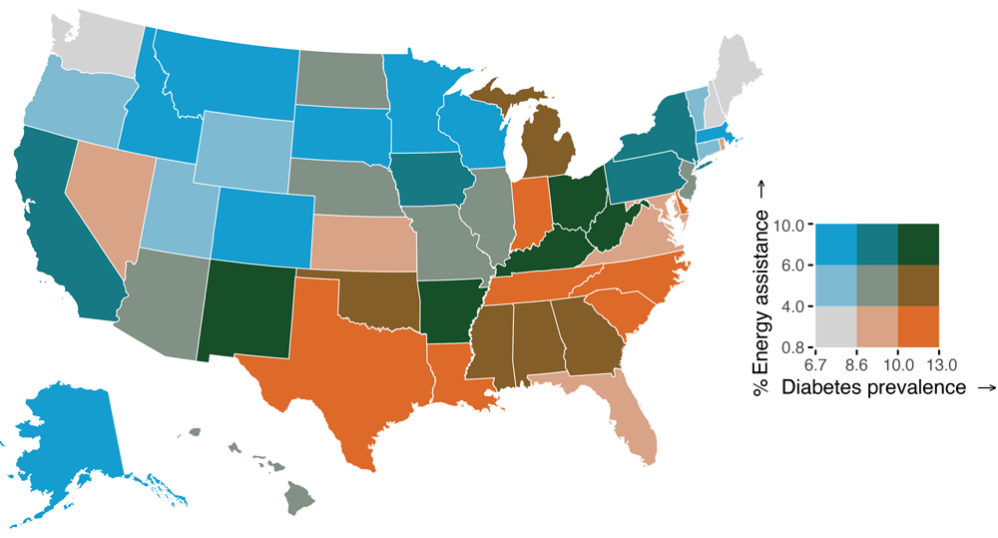
Bivariate map of the age-standardized percentage of ever receiving energy assistance and diagnosed diabetes prevalence by US states, 2020. Note: Cutoffs of ever receiving energy assistance and diabetes prevalence were established based on tertiles. Sources: 2020 Residential Energy Consumption Survey (RECS) (6); 2020 Centers for Disease Control and Prevention’s US Diabetes Surveillance System.

The crude prevalence of any household energy insecurity among an estimated 123.5 million US households was 27.2% (95% CI, 26.4–28.0; range, 14.7% in Vermont to 40.4% in Mississippi), 19.9% (95% CI, 19.2–20.6) for reducing or forgoing food or medicine to pay energy costs, 9.9% (95% CI, 9.3–10.5) for leaving home at unhealthy temperatures, 10.0% (95% CI, 9.5–10.5) for receiving a disconnect or delivery stop notice, 4.0% (95% CI, 3.6–4.4) for being unable to use heating equipment, and 5.1% (95% CI, 4.7–5.5) for being unable to use air conditioning equipment (Table). The prevalence of ever receiving energy assistance was 5.3% (95% CI, 4.9–5.7; range, 3.1% in Virginia to 10.0% in California), while in 2020 alone, 3.5% (95% CI, 3.2–3.8) of US households received energy assistance (data not shown). The age-standardized bivariate choropleth map revealed that states with a higher percentage of energy insecurity also had a greater diagnosed diabetes prevalence, compared with states with lower levels of energy insecurity (Figure 1). The highest prevalence of any household energy insecurity and diabetes was found mostly in southern states (Alabama, Arkansas, Georgia, Kentucky, Louisiana, Mississippi, North Carolina, Oklahoma, South Carolina, Texas, and West Virginia), as well as Indiana and Michigan. Similarly, the adjusted linear regression model showed a positive association between household energy insecurity and diagnosed diabetes prevalence (b = 0.17, 95% CI, 0.11–0.24, *P* < .001) (data not shown). Furthermore, the states with the lowest prevalence of ever receiving energy assistance and the highest diabetes prevalence were Indiana and southern states that include Louisiana, North Carolina, South Carolina, Tennessee, and Texas (Figure 2).

**Table:** Crude Prevalence of Energy Insecurity Measures and Receipt of Energy Assistance by State, 2020 Residential Energy Consumption Survey

State	Any household energy insecurity,^a^ % (95% CI)	Reducing or forgoing food or medicine to pay energy costs, % (95% CI)	Leaving the home at unhealthy temperature, % (95% CI)	Receiving disconnect or delivery stop notice, % (95% CI)	Unable to use heating equipment, % (95% CI)	Unable to use air conditioning equipment, % (95% CI)	Ever received energy assistance, % (95% CI)
Total	27.2 (26.4–28.0)	19.9 (19.2–20.6)	9.9 (9.3–10.5)	10.0 (9.5–10.5)	4.0 (3.6–4.4)	5.1 (4.7–5.5)	5.3 (4.9–5.7)
Alabama	33.7 (27.6–39.7)	27.2 (21.7–32.7)	14.6 (9.3–19.9)	11.2 (6.8–15.6)	7.0 (3.7–10.3)	8.7 (5.0–12.3)	3.5 (0.9–6.0)
Alaska	24.9 (20.0–29.9)	17.4 (13.3–21.6)	10.3 (6.7–13.9)	11.2 (6.6–15.7)	4.6 (2.0–7.3)	—b	7.9 (5.0–10.9)
Arizona	26.8 (22.9–30.7)	19.9 (16.0–23.7)	11.2 (8.3–14.2)	8.2 (5.8–10.6)	4.9 (2.9–7.0)	6.5 (4.2–8.9)	4.3 (2.1–6.6)
Arkansas	36.2 (30.0–42.4)	26.2 (20.5–31.9)	11.2 (6.3–16.0)	15.1 (10.3–19.9)	8.4 (5.0–11.9)	10.2 (6.3–14.0)	6.6 (2.7–10.4)
California	30.5 (27.8–33.3)	20.9 (18.4–23.4)	13.8 (11.7–15.8)	7.1 (5.3–8.9)	4.7 (3.4–6.0)	5.8 (4.2–7.3)	10.0 (8.1–11.8)
Colorado	23.6 (19.2–28.1)	18.3 (14.4–22.3)	7.7 (4.6–10.8)	8.0 (4.7–11.3)	3.0 (1.0–5.1)	3.8 (1.0–6.6)	5.8 (3.1–8.5)
Connecticut	27.0 (22.0–32.0)	19.7 (15.2–24.2)	13.3 (8.3–18.3)	9.2 (5.7–12.7)	5.4 (2.5–8.4)	5.3 (2.5–8.1)	5.9 (2.2–9.6)
Delaware	25.7 (17.9–33.5)	19.1 (12.5–25.7)	8.5 (3.7–13.3)	8.3 (3.7–12.9)	3.3 (0.1–6.4)	4.1 (0.8–7.3)	—b
District of Columbia	18.3 (12.7–23.8)	13.4 (8.4–18.4)	6.2 (3.1–9.3)	6.3 (2.5–10.0)	4.2 (1.3–7.1)	3.2 (0.8–5.6)	3.4 (0.6–6.2)
Florida	22.6 (19.4–25.7)	17.9 (14.9–20.9)	7.1 (4.6–9.5)	8.7 (6.1–11.3)	3.7 (2.2–5.2)	6.1 (4.0–8.3)	—b
Georgia	33.5 (28.9–38.1)	23.8 (19.2–28.5)	12.4 (8.4–16.3)	15.7 (12.0–19.4)	5.3 (2.6–7.9)	9.1 (6.3–11.9)	6.0 (3.4–8.5)
Hawaii	23.6 (18.5–28.6)	15.7 (11.2–20.1)	7.3 (4.1–10.4)	6.0 (2.7–9.3)	2.5 (0.5–4.6)	3.5 (1.2–5.8)	5.2 (2.0–8.3)
Idaho	19.3 (14.9–23.6)	14.2 (10.0–18.4)	7.1 (4.1–10.1)	7.1 (3.6–10.7)	1.5 (0.1–3.0)	3.4 (1.2–5.6)	7.4 (3.5–11.3)
Illinois	23.5 (19.3–27.6)	18.0 (14.3–21.7)	7.5 (4.9–10.1)	7.4 (4.7–10.1)	2.6 (1.1–4.1)	4.3 (2.4–6.2)	6.2 (3.8–8.5)
Indiana	28.9 (24.2–33.6)	22.4 (18.3–26.5)	8.5 (5.5–11.6)	14.9 (11.3–18.6)	4.8 (2.5–7.2)	5.1 (2.7–7.5)	3.3 (1.3–5.4)
Iowa	17.7 (13.0–22.3)	14.8 (10.3–19.3)	4.1 (1.5–6.8)	6.8 (3.7–9.9)	—b	—b	7.1 (3.5–10.6)
Kansas	25.4 (19.6–31.3)	15.2 (9.6–20.7)	8.1 (4.2–12.1)	8.4 (4.3–12.4)	2.7 (0.3–5.1)	4.5 (1.5–7.4)	—b
Kentucky	32.9 (27.1–38.8)	22.3 (17.3–27.2)	9.7 (6.5–12.9)	16.8 (12.3–21.3)	3.9 (2.0–5.8)	6.8 (4.1–9.4)	6.0 (3.6–8.3)
Louisiana	33.2 (28.2–38.2)	26.0 (21.0–31.1)	10.3 (7.1–13.6)	15.6 (11.4–19.8)	3.9 (1.8–6.0)	8.0 (4.7–11.3)	4.0 (1.3–6.8)
Maine	23.1 (17.4–28.8)	16.5 (11.0–22.0)	8.3 (4.2–12.3)	10.3 (4.9–15.8)	7.5 (3.6–11.4)	3.9 (1.2–6.6)	3.9 (1.3–6.4)
Maryland	22.5 (18.2–26.8)	16.7 (12.6–20.8)	9.0 (5.8–12.1)	10.0 (6.8–13.3)	2.8 (0.8–4.7)	4.0 (1.6–6.5)	3.9 (1.5–6.3)
Massachusetts	22.2 (17.4–27.1)	15.1 (10.4–19.7)	9.3 (6.4–12.2)	5.1 (3.0–7.1)	2.4 (1.0–3.8)	1.8 (0.7–3.0)	8.1 (5.2–11.0)
Michigan	29.4 (24.4–34.3)	20.9 (16.6–25.2)	9.1 (5.9–12.3)	10.9 (7.7–14.2)	3.8 (1.9–5.8)	2.6 (0.8–4.5)	5.1 (2.7–7.5)
Minnesota	16.5 (12.2–20.8)	12.6 (8.6–16.6)	4.0 (1.9–6.0)	5.3 (2.8–7.7)	—b	1.7 (0.3–3.1)	7.0 (3.9–10.2)
Mississippi	40.4 (32.0–48.8)	33.1 (25.5–40.8)	13.3 (8.4–18.2)	14.8 (8.6–20.9)	6.3 (2.4–10.3)	10.5 (6.2–14.7)	4.1 (0.3–8.0)
Missouri	26.8 (22.1–31.5)	21.1 (16.7–25.5)	9.3 (5.5–13.1)	15.6 (10.8–20.5)	8.5 (5.0–11.9)	6.8 (3.9–9.7)	5.1 (2.3–8.0)
Montana	24.4 (17.0–31.8)	18.3 (10.9–25.7)	7.8 (3.6–12.0)	9.0 (4.3–13.7)	—b	3.2 (0.3–6.2)	7.9 (2.9–13.0)
Nebraska	16.8 (10.3–23.3)	11.8 (6.2–17.4)	5.6 (1.5–9.7)	7.1 (3.1–11.0)	3.3 (0.5–6.1)	—b	3.8 (0.4–7.2)
Nevada	29.4 (22.0–36.9)	22.1 (15.8–28.4)	13.1 (7.3–18.9)	9.4 (5.0–13.7)	6.1 (1.7–10.5)	6.5 (2.6–10.5)	3.7 (0.3–7.0)
New Hampshire	22.6 (15.7–29.5)	12.2 (6.3–18.1)	5.6 (1.8–9.5)	9.6 (4.9–14.3)	3.0 (0.3–5.6)	5.2 (1.9–8.5)	3.1 (0.3–5.9)
New Jersey	25.3 (21.0–29.6)	18.4 (14.5–22.3)	8.6 (5.3–12.0)	10.2 (7.2–13.2)	2.7 (1.1–4.3)	4.9 (2.7–7.1)	5.3 (3.0–7.7)
New Mexico	25.4 (19.1–31.7)	15.7 (9.7–21.8)	11.5 (6.2–16.8)	5.7 (1.9–9.5)	4.3 (0.7–7.9)	3.2 (0.4–6.1)	7.5 (3.1–11.8)
New York	27.8 (24.8–30.8)	18.3 (15.8–20.9)	12.7 (10.3–15.0)	9.1 (7.0–11.2)	3.3 (1.9–4.8)	4.5 (2.8–6.2)	6.3 (4.5–8.0)
North Carolina	27.5 (23.3–31.6)	20.4 (16.2–24.6)	9.4 (6.3–12.5)	11.6 (8.3–15.0)	3.3 (1.3–5.2)	4.6 (2.7–6.6)	3.6 (1.8–5.4)
North Dakota	21.4 (15.7–27.0)	14.2 (9.4–18.9)	9.1 (5.3–12.9)	9.7 (6.2–13.2)	2.0 (0.4–3.6)	4.4 (1.8–7.0)	4.3 (1.8–6.9)
Ohio	26.1 (21.5–30.8)	18.2 (14.3–22.2)	7.4 (4.4–10.5)	15.3 (11.1–19.5)	4.1 (1.7–6.4)	4.4 (2.1–6.6)	6.9 (3.8–10.1)
Oklahoma	34.2 (28.0–40.5)	25.9 (20.2–31.6)	12.0 (6.5–17.5)	17.1 (11.7–22.5)	8.9 (4.5–13.3)	10.7 (6.3–15.1)	5.6 (2.3–8.9)
Oregon	21.4 (16.2–26.6)	16.7 (12.0–21.4)	9.3 (5.3–13.3)	3.7 (1.4–6.0)	3.0 (0.9–5.1)	3.6 (1.2–5.9)	3.3 (1.2–5.5)
Pennsylvania	23.9 (20.1–27.8)	17.3 (13.8–20.9)	11.0 (8.2–13.7)	7.8 (5.5–10.1)	3.8 (2.2–5.5)	2.9 (1.6–4.2)	5.7 (3.4–8.0)
Rhode Island	23.3 (15.6–31.0)	15.4 (9.3–21.5)	10.4 (4.7–16.0)	7.0 (2.3–11.7)	9.4 (4.8–14.0)	3.7 (0.3–7.2)	4.1 (1.3–7.0)
South Carolina	32.1 (26.9–37.3)	26.4 (21.6–31.2)	10.7 (6.9–14.5)	14.5 (10.6–18.5)	5.0 (2.8–7.2)	7.2 (4.1–10.4)	3.2 (1.3–5.2)
South Dakota	20.1 (12.5–27.8)	16.1 (9.2–23.0)	6.9 (2.0–11.8)	6.3 (2.4–10.2)	—b	2.3 (0.0–4.6)	6.7 (2.6–10.7)
Tennessee	27.5 (23.7–31.3)	22.0 (18.2–25.8)	9.8 (7.0–12.6)	11.7 (8.6–14.8)	4.1 (2.0–6.2)	6.9 (4.3–9.5)	4.0 (2.3–5.7)
Texas	34.5 (31.2–37.8)	26.2 (23.1–29.4)	10.2 (8.0–12.5)	13.0 (10.8–15.3)	4.4 (3.3–5.4)	6.0 (4.3–7.7)	3.3 (2.1–4.6)
Utah	19.1 (12.8–25.5)	11.9 (6.3–17.6)	4.6 (1.1–8.2)	9.0 (4.2–13.8)	5.2 (1.2–9.2)	4.9 (1.5–8.3)	3.6 (0.3–6.9)
Vermont	14.7 (10.4–19.0)	11.7 (7.6–15.9)	5.0 (2.3–7.7)	4.7 (2.2–7.2)	4.7 (2.1–7.3)	—b	4.4 (1.8–7.0)
Virginia	24.7 (20.4–28.9)	17.8 (14.0–21.5)	8.3 (5.4–11.2)	7.3 (5.0–9.6)	2.9 (1.2–4.6)	3.7 (2.0–5.4)	3.1 (1.2–5.0)
Washington	22.0 (17.3–26.6)	15.2 (11.0–19.4)	9.2 (5.9–12.5)	7.5 (4.3–10.7)	1.5 (0.3–2.6)	2.1 (0.7–3.6)	3.7 (1.9–5.6)
West Virginia	37.3 (30.4–44.2)	30.4 (24.0–36.8)	14.4 (8.9–19.8)	14.3 (9.3–19.3)	7.5 (3.6–11.4)	11.8 (6.7–16.9)	5.9 (2.6–9.2)
Wisconsin	20.5 (15.6–25.3)	15.6 (11.3–19.8)	6.6 (3.6–9.6)	4.7 (2.6–6.7)	1.3 (0.1–2.4)	3.6 (1.2–6.0)	9.1 (5.5–12.6)
Wyoming	20.7 (13.8–27.6)	14.0 (8.3–19.6)	6.7 (2.9–10.5)	7.6 (3.1–12.1)	3.6 (0.5–6.8)	—b	6.0 (2.5–9.4)

a Defined as having 1 of these 5 experiences: having to reduce or forgo food or medicine to pay energy costs, leaving the home at unhealthy temperatures, receiving disconnect or delivery stop notice, being unable to use heating equipment due to cost, or being unable to use air-conditioning equipment because of cost.

b Suppressed because of a relative standard error >50%.

## Discussion

Overall, states with a higher prevalence of household energy insecurity had a higher prevalence of diagnosed diabetes, with the highest prevalence of both concentrated mainly among southern states. Diabetes prevalence has continued to increase for people with low incomes (13). These trends, overlaid with more extreme temperature events over the past several decades because of climate change, indicate a burgeoning crisis (1). Additionally, we found that reducing or forgoing food or medicine to pay energy costs was the most common form of energy insecurity. This may contribute to challenges with diabetes management (eg, insulin rationing) and increases in diabetes-related complications (14).

The low prevalence of ever receiving energy assistance highlights an opportunity to reduce energy insecurity in states with high diabetes burden. Federal policies such as the Low-Income Home Energy Assistance Program and the Weatherization Assistance Program provide financial support to families with low incomes for energy bill payments, weatherization, and energy-related home repairs (4). However, these programs have been persistently underfunded and subject to budget cuts, undermining critical access to energy-related assistance programs for low-income households (4). State policies and utility companies may also address energy insecurity, as some states have policies prohibiting utility companies from disconnecting gas or electricity for households with people who have or are at greater risk for medical conditions (eg, diabetes) or have seasonal policies that forbid disconnections during extreme weather (4). The drawback to these policies is that many are time-limited and may not adequately address the needs of people with chronic household energy insecurity. At the local level, implementation of cooling centers has shown promise in sheltering high-risk populations from extreme heat and providing heat safety education, but residents may not be aware of or have access to these resources (15). At the clinic level, screening patients with diabetes for energy insecurity and referring them to state and community level resources for energy assistance would be important given that clinical interventions addressing social needs can improve health outcomes, reduce health care costs, and increase preventive care utilization (16). Future research could examine how to better implement these various policies and interventions and their effect on diabetes outcomes.

Limitations of this study include 1) household energy and diabetes are self-reported, resulting in misclassification bias, and 2) state-level associations may not apply at the individual level. Notwithstanding these limitations, developing new policies and strengthening existing ones could help to reduce household energy insecurity and subsequently decrease disparities in diabetes-related outcomes.
